# Diagnostic Performance of Preoperative Multiparametric MRI for Local and Nodal Staging in Penile Cancer: A Histopathology-Correlated Single-Centre Study

**DOI:** 10.3390/cancers18162674

**Published:** 2026-08-18

**Authors:** Mateusz Czajkowski, Michał Falis, Jan Mandrysz, Oliwia Kozak, Karolina Markiet, Marcin Markuszewski, Agnieszka Rybarczyk, Piotr M. Wierzbicki, Marcin Matuszewski, Oliver W. Hakenberg

**Affiliations:** 1 Department of Urology, Medical University of Gdańsk, Mariana Smoluchowskiego 17 Street, 80-214 Gdańsk, Poland; marcin.markuszewski@gumed.edu.pl (M.M.); marcin.matuszewski@gumed.edu.pl (M.M.); 2 Student Scientific Circle at the Department of Urology, Faculty of Medicine, Medical University of Gdańsk, Marii Skłodowskiej-Curie 3a, 80-210 Gdańsk, Poland; mfalis@uck.gda.pl; 3 Division of Radiology Informatics and Statistics, Medical University of Gdańsk, Tuwima 15 Street, 80-210 Gdańsk, Poland; jan.mandrysz@gumed.edu.pl; 4 Department of Radiology, Medical University of Gdańsk, Mariana Smoluchowskiego 17 Street, 80-214 Gdańsk, Poland; oliwia.kozak@gumed.edu.pl (O.K.);; 5 Department of Histology, Medical University of Gdańsk, Dębinki 1, 80-211 Gdańsk, Poland; agnieszka.rybarczyk@gumed.edu.pl (A.R.); pwierzb@gumed.edu.pl (P.M.W.); 6 Department of Urology, Jena University Hospital, 07747 Jena, Germany; oliver.hakenberg@med.uni-jena.de

**Keywords:** penile cancer, multiparametric magnetic resonance imaging, local staging, nodal staging, diagnostic accuracy, histopathology, lymph node metastasis

## Abstract

**Background/Objectives**: Histopathology-correlated evidence for preoperative magnetic resonance imaging (MRI) in penile cancer remains limited. We evaluated multiparametric MRI with artificially induced erection for local and nodal staging in surgically treated penile cancer. **Methods**: This prospective, single-centre diagnostic accuracy study included consecutive patients who underwent preoperative multiparametric MRI between 2017 and 2024, with postoperative histopathology as the reference standard. The primary endpoint was binary local T-staging (≤T1 vs. ≥T2). Secondary endpoints were three-category T-staging (≤T1/T2/≥T3) and binary nodal staging (N0 vs. N+) in patients with pathological nodal verification. Two radiologists independently reassessed all 38 examinations in a post hoc reproducibility analysis. **Results**: Thirty-eight patients were included; all tumours were glans-based and treated with organ-sparing surgery. Binary local staging showed 68.4% accuracy (95% CI, 52.5–80.9%), 88.2% sensitivity, 52.4% specificity, 60.0% positive predictive value, 84.6% negative predictive value, and balanced accuracy of 0.703 (95% CI, 0.568–0.838). Overstaging predominated (10 false-positive vs. 2 false-negative classifications; *p* = 0.043). Three-category exact agreement was 52.6% (κ = 0.257). In the pathologically verified nodal subset (*n* = 30), accuracy was 83.3% (95% CI, 66.4–92.7%), sensitivity 78.6%, specificity 87.5%, and balanced accuracy 0.830 (95% CI, 0.691–0.970). Inter-reader agreement was 92.1% for binary T staging (κ = 0.837) and 97.4% for binary N staging (κ = 0.943). **Conclusions**: mpMRI was sensitive but insufficiently specific for ≥T2 disease and should not independently trigger treatment escalation. Nodal performance was encouraging but arose from a guideline-selected verified subset. These findings apply primarily to glans-based, organ-preserving cohorts and do not establish an incremental benefit of erection induction.

## Simple Summary

Penile cancer treatment depends on tumour depth and lymph node involvement. Multiparametric magnetic resonance imaging (mpMRI) may supplement clinical staging, but histopathology-correlated evidence remains limited. We studied 38 men with penile cancer who underwent this MRI before definitive surgery; all tumours were glans-based, and all patients underwent organ-sparing treatment. MRI detected most tumours that were pathologically classified as ≥T2 but frequently overstaged tumours confined to ≤T1. In the 30 patients with complete pathological nodal verification, MRI showed encouraging performance for distinguishing N0 from N+ disease; the remaining eight patients were clinically node-negative and did not undergo invasive nodal staging because their primary tumours met EAU low-risk criteria. A post hoc two-reader analysis showed high inter-reader agreement. These findings support mpMRI as an adjunct in selected staging and surgical-planning dilemmas. MRI should not replace clinical examination, histopathology, dynamic sentinel node biopsy when indicated, or therapeutic lymphadenectomy.

## 1. Introduction

Penile cancer is a rare malignancy whose epidemiology is closely correlated with the degree of regional industrialisation [1,2]. The overall incidence in Europe is approximately 0.94 per 100,000 males and 0.5 per 100,000 in the United States of America (USA), whereas in South America, Southeast Asia, and parts of Africa, penile cancer may account for up to 1–2% of all male malignancies [3,4]. The primary risk factors include phimosis, lack of circumcision, human papillomavirus (HPV) infection, cigarette smoking, obesity, and rural residence [5,6]. Due to the intimate anatomical location of the lesions, patients frequently delay seeking medical attention as a result of embarrassment and limited awareness of the disease’s symptoms and risk factors, which may result in a more advanced disease at the time of diagnosis [7,8]. Accurate preoperative local and nodal staging are the most critical determinants of treatment selection and prognosis. According to the current EAU-ASCO guidelines, the presence and extent of lymph node metastases are the most important prognostic factors for survival, with 5-year cancer-specific survival rates of approximately 95%, 80%, 65%, and 35% for N0, N1, N2, and N3 disease, respectively [9,10,11]. However, preoperative assessment of local tumour extent and nodal status remains challenging. Accurate nodal staging is particularly difficult in cN0 disease because conventional imaging modalities cannot detect micrometastatic disease, which may significantly impact treatment decisions and survival outcomes [12,13].

Physical examination remains the primary diagnostic tool in the evaluation of penile cancer and should include inspection and palpation of the entire penis to identify potential skip lesions, as well as precise documentation of lesion size, anatomical location, extent of local invasion, stretched penile length, and clinical evaluation of the inguinal lymph nodes [12]. However, a fundamental limitation of physical examination is its inability to accurately determine the depth of tumour infiltration into the underlying penile structures preoperatively. Magnetic resonance imaging (MRI), owing to its superior soft-tissue resolution, offers a potential solution to this diagnostic challenge [14]. Nevertheless, for early-stage disease (T1–T2), MRI does not outperform physical examination [12]. According to current guidelines, MRI is indicated when there is uncertainty regarding cavernosal body invasion (cT3), when organ-sparing surgery is being considered, and prior to the resection of large tumours clinically staged as T4 [12].

Available data on the diagnostic performance of MRI for penile cancer remain limited, heterogeneous, and predominantly derived from small, single-centre cohorts [15,16]. A 2022 systematic review and meta-analysis by Krishna et al. evaluated the diagnostic performance of MRI across multiple clinical staging scenarios, reporting a pooled sensitivity of 86% and specificity of 89% for distinguishing ≤T1 from ≥T2 disease; however, this meta-analysis did not demonstrate the superiority of MRI over clinical staging [17]. The strongest diagnostic performance of MRI was observed in the detection of corporal invasion (≥T3), with a pooled sensitivity of 80% (95% CI: 70–87%) and specificity of 96% (95% CI: 85–99%) [17]. Notably, the same meta-analysis found that MRI performed with and without prostaglandin E1-induced artificial erection yielded similar accuracy in local T-staging, suggesting that artificially induced erection may not confer a meaningful diagnostic advantage [17].

In this context, further validation of preoperative MRI in penile cancer through histopathological analysis remains necessary. The present study assessed the diagnostic performance and error pattern of a standardised preoperative mpMRI protocol, which included prostaglandin E1-induced erection, in patients undergoing surgical treatment for penile cancer. Postoperative histopathology served as the reference standard. The primary objective was to assess the ability of MRI to distinguish ≤T1 from ≥T2 local disease. Secondary objectives were to evaluate agreement across the three-category classification of ≤T1, T2, and ≥T3 disease and to assess binary nodal staging in patients with complete pathological nodal verification. A post hoc two-reader reassessment was additionally performed to evaluate inter-reader reproducibility. The influence of equivocal T1/T2 classifications was examined in an exploratory sensitivity analysis. The study was not designed to compare MRI with and without pharmacologically induced erection or to establish superiority over clinical examination.

## 2. Materials and Methods

### 2.1. Study Design, Patients, and Endpoints

This was a single-centre diagnostic-accuracy analysis of a prospectively collected cohort. The analytic cohort comprised all consecutive eligible patients who underwent preoperative mpMRI as part of routine clinical work-up and subsequent definitive surgical treatment between 2017 and 2024. The decision to obtain an MRI was made during routine clinical care and was not assigned by the study protocol. Accordingly, the cohort represents consecutive MRI-examined surgical patients rather than all patients with penile cancer treated at the centre during the study period. No additional sampling was performed within the eligible MRI-examined cohort. Final postoperative histopathology served as the reference standard. The study was approved by the university Ethics Committee (Decision No. NKBBN/369/2017); the approval was subsequently extended and expanded under Decision No. NKBBN/168/2023. Written informed consent was obtained from all participants before surgery. Histological subtypes were classified according to the 2022 World Health Organization classification [18]. p16 immunohistochemistry was recorded as a surrogate marker of HPV-associated disease.

**Figure 1:**
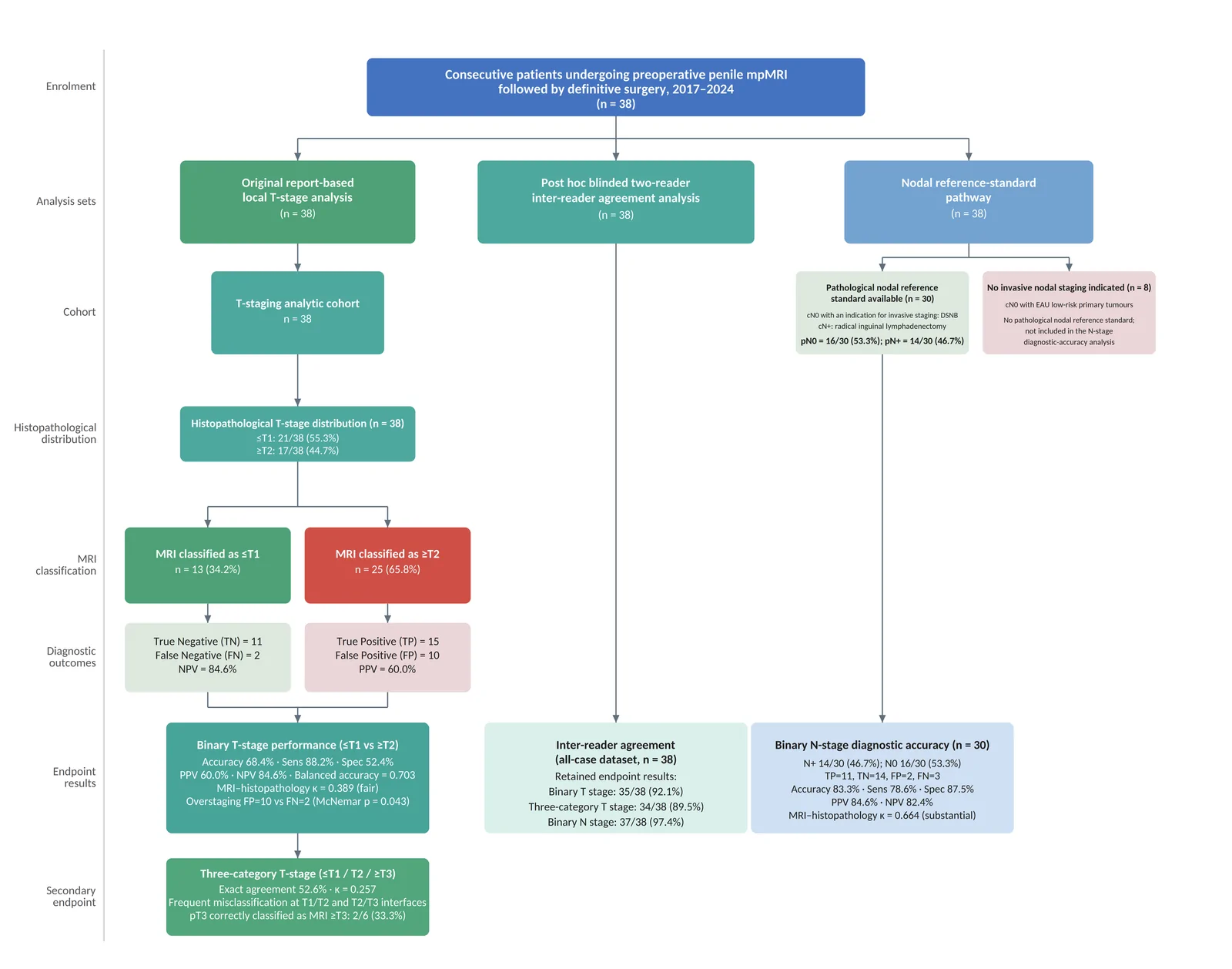
Study flow diagram and endpoint-specific analysis sets. The primary local T-stage diagnostic-accuracy analyses were based on the original preoperative MRI reports and included all 38 patients. A separate post hoc blinded two-reader inter-reader agreement analysis was performed using the full cohort (*n* = 38). A pathological nodal reference standard was available in 30 patients and was established by dynamic sentinel node biopsy in cN0 patients with a guideline-based indication for invasive nodal staging and by radical inguinal lymphadenectomy in cN+ patients. In eight cN0 patients with EAU low-risk primary tumours, invasive nodal staging was not indicated; therefore, no pathological nodal reference standard was available, and these patients were not included in the N-stage diagnostic-accuracy analysis. DSNB, dynamic sentinel node biopsy; MRI, magnetic resonance imaging; NPV, negative predictive value; PPV, positive predictive value.

Eligibility for the present analysis required preoperative multiparametric MRI, definitive surgical treatment, and available final postoperative histopathology for local T-stage assessment. Patients with MRI performed after neoadjuvant treatment, incomplete postoperative histopathology, histology other than squamous penile neoplasia, or insufficient image quality for staging were excluded. All included patients underwent definitive surgery within one month after the MRI (Figure 1). Diagnostic biopsy or cytological sampling was performed as part of routine clinical care. When sampling preceded MRI, the exact sampling-to-imaging interval was not systematically recorded; therefore, the potential effect of post-procedural haemorrhage, oedema, inflammation, or local architectural distortion on MRI interpretation could not be formally evaluated.

The prespecified endpoints were as follows:

1. Binary T-stage: early (≤T1) versus advanced (≥T2).
2. Three-category T stage: ≤T1, T2, and T3+ (advanced).
3. Binary N-stage: N0 versus N+ in patients with complete pathological nodal verification, defined as DSNB in cN0 patients and radical inguinal lymphadenectomy in cN+ patients.

In addition to the prespecified endpoints, a post hoc independent two-reader reassessment was performed to evaluate inter-reader reproducibility of local and nodal MRI staging.

### 2.2. Index Test (MRI) and Staging Classification

**Figure 2:**
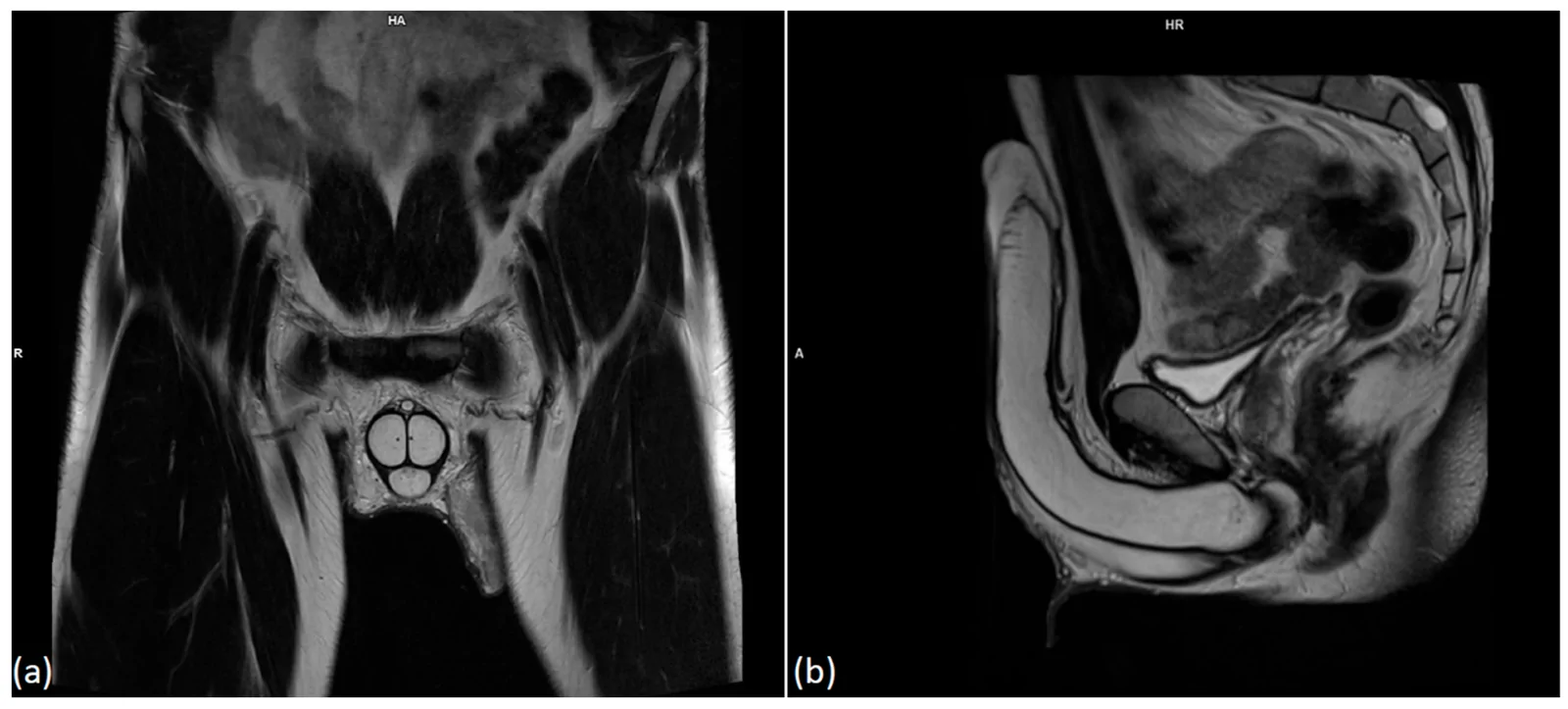
Normal penile anatomy on T2-weighted MRI (artificially induced erection): (**a**) coronal and (**b**) sagittal images delineating the penile structures.

The index test was a preoperative mpMRI performed as part of a routine clinical work-up. Pharmacologically induced erection was a standardized component of the institutional acquisition protocol (Figure 2). Because no examination without erection was available, the study was not designed to assess the incremental diagnostic contribution of erection induction. MRI stage categories were limited to those assessable by imaging (T0, Tis, T1, T2, T3, and T4).

Immediately before image acquisition, prostaglandin E1 was administered intracavernosally at a dose of 10–20 μg under joint urological and radiological supervision to induce an erection. Preoperative multiparametric MRI was performed on a 1.5-T scanner (Aera; Siemens, Erlangen, Germany) using a multichannel phased-array surface coil.

The acquisition protocol comprised high-resolution T2-weighted imaging of the penis in the transverse, sagittal, and coronal planes (repetition time [TR]/echo time [TE], 5660/75 ms; field of view [FOV], 280 × 280 mm; matrix, 320 × 320; slice thickness, 3.0 mm), T1-weighted imaging (TR/TE, 550/19 ms; FOV, 320 × 320 mm; matrix, 320 × 256; slice thickness, 3.0 mm), and diffusion-weighted imaging (DWI; TR/TE, 9800/68 ms; FOV, 400 × 400 mm; matrix, 120 × 160; slice thickness, 3.5 mm) acquired at b values of 50, 500, 800, and 1200 s/mm^2^. Apparent diffusion coefficient maps were derived from the DWI data.

Dynamic contrast-enhanced (DCE) imaging was performed with coverage extending through the pelvis to the aortic bifurcation after intravenous administration of a gadolinium-based contrast agent (Gadovist; Bayer, Berlin, Germany) at a dose of 0.1 mL/kg. The DCE protocol comprised eight consecutive fat-suppressed T1-weighted dynamic series acquired in 30 s cycles (TR/TE, 3.99/1.56 ms; FOV, 330 × 330 mm; matrix, 192 × 154; slice thickness, 3.5 mm), with subtraction images generated for interpretation.

The index test was the local T category and nodal N category assigned in the original preoperative MRI report. All examinations were interpreted by a single experienced genitourinary radiologist who was a member of the genitourinary imaging group of the European Society of Urogenital Radiology (ESUR) (O.K.). The radiologist was aware of the clinical diagnosis of penile cancer but was blinded to the final postoperative histopathological findings at the time of image interpretation. The MRI T and N categories used in the analyses were extracted from the original preoperative reports; no retrospective rereading or post hoc modification of the MRI stage was performed after the histopathological results became available.

A key methodological constraint was that MRI could not reliably determine the microscopic features required for the histopathologic subclassification of T1 disease (e.g., lymphovascular invasion, perineural invasion, and grade). Therefore, T1a and T1b could not be distinguished on MRI, and all MRI T1 lesions were classified as T1.

**Figure 3:**
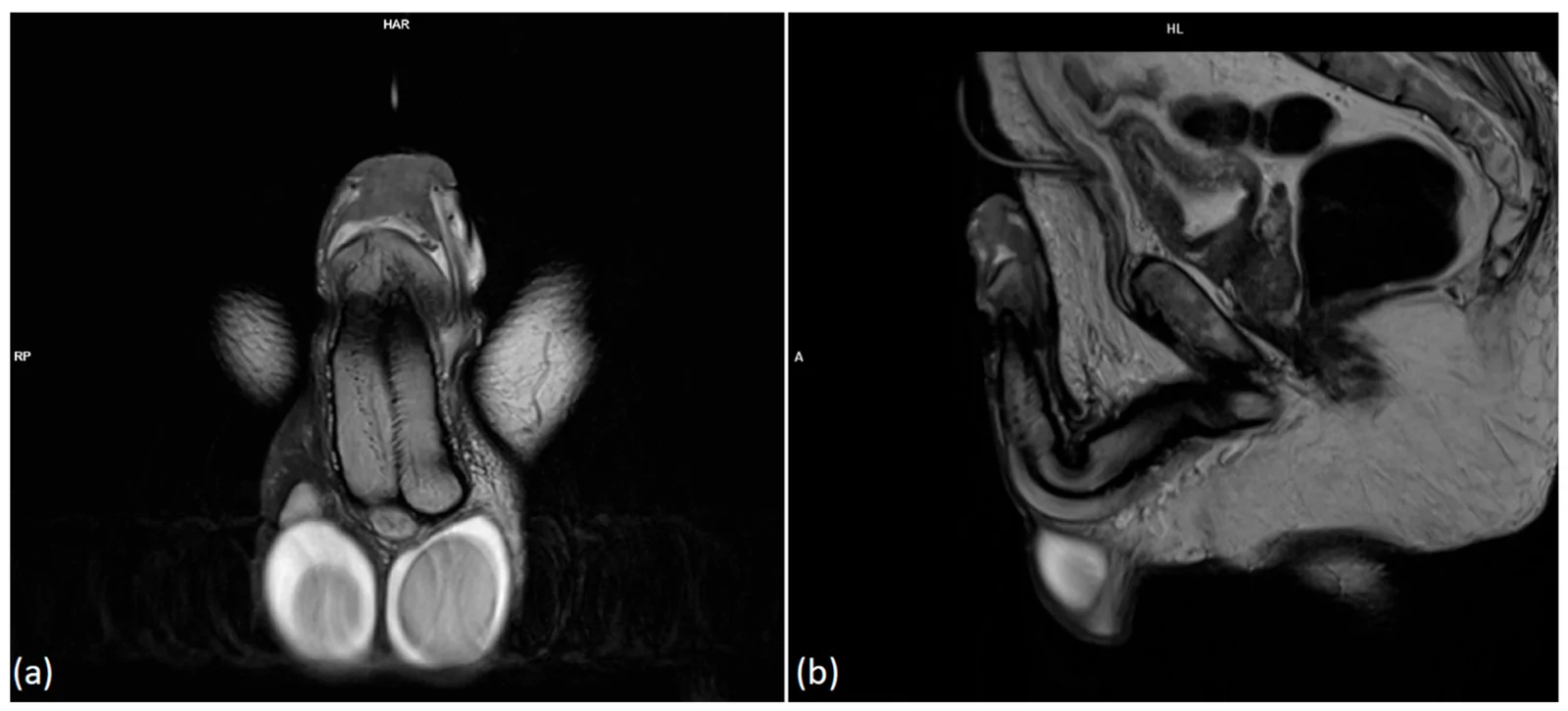
Tumour confined to the epithelium or subepithelial tissue of the glans without radiological evidence of corpus spongiosum or corpus cavernosum invasion (≤T1): (**a**) axial and (**b**) sagittal T2-weighted images.

**Figure 4:**
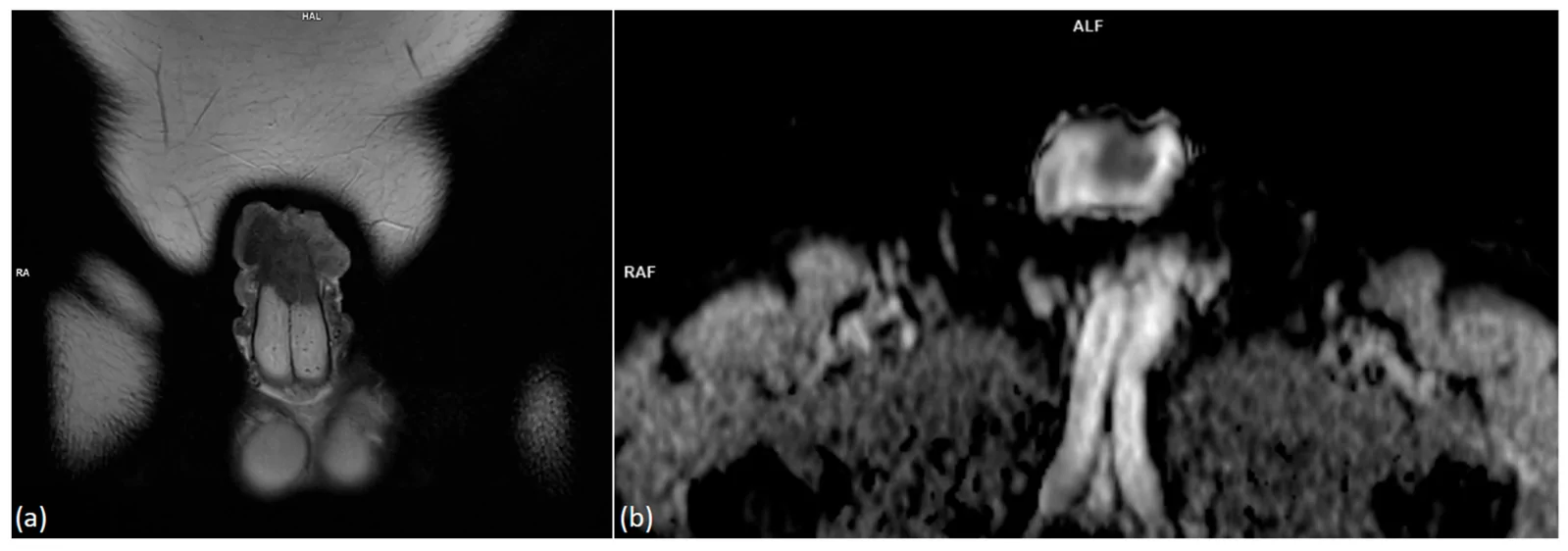
Corpus cavernosum invasion (T3): (**a**) coronal T2-weighted image; (**b**) corresponding restricted diffusion on ADC map.

**Figure 5:**
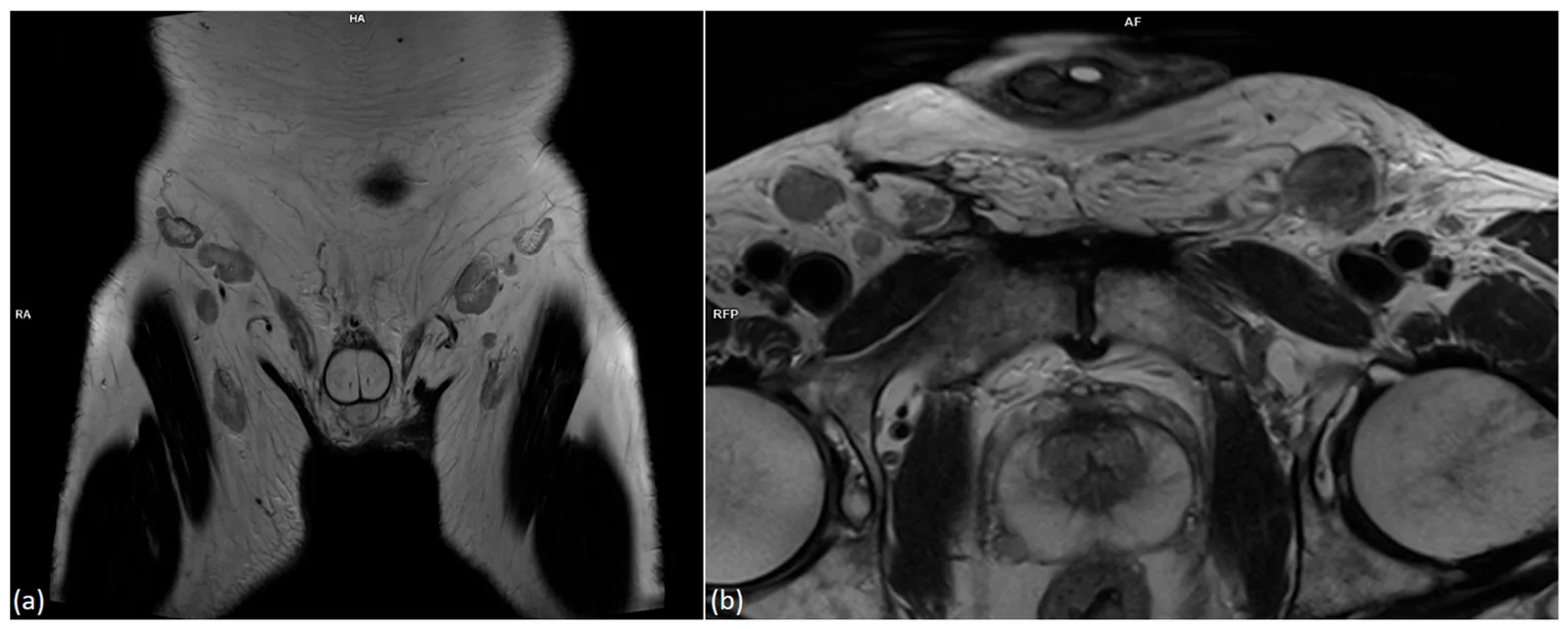
Inguinal lymph node assessment using MRI: (**a**) morphologically suspicious inguinal nodes that were pathologically negative, representing a false-positive MRI finding; (**b**) pathologically metastatic inguinal nodes, which may be non-palpable when small.

For MRI-based local staging, the T-category was assigned based on the deepest penile anatomical structure suspected of tumour invasion. Disease confined to the epithelium or subepithelial tissue without radiological evidence of corpus spongiosum or corpus cavernosum invasion was classified as ≤T1 (Figure 3). Suspected invasion of the corpus spongiosum was classified as T2, while suspected invasion of the corpus cavernosum was classified as T3 (Figure 4). Suspected invasion of adjacent structures was classified as T4. For nodal staging, MRI findings were categorised as N0 or N+ according to the presence of morphologically suspicious inguinal or pelvic lymph nodes, with assessment of size, shape, border irregularity, internal architecture, necrosis, diffusion restriction, and radiological evidence of extranodal extension (Figure 5).

For the binary T-stage analysis, the MRI results were dichotomised as ≤T1 (negative) versus ≥T2 (positive). For the three-category analysis, the MRI and histopathological categories were expanded to ≤T1, T2, and ≥T3.

### 2.3. Post Hoc Two-Reader Methods

To evaluate inter-reader reproducibility, two radiologists with experience in genitourinary imaging (O.K. and K.M.) independently reassessed all 38 MRI examinations using a structured staging form. Each reader assigned a binary local T category (≤T1 vs. ≥T2), a three-category local T category (≤T1, T2, or ≥T3), and a binary nodal category (N0 vs. N+). The reassessment was conducted as a post hoc secondary analysis and did not alter the original report-based classifications used for the primary diagnostic-accuracy endpoints. Both readers were blinded to the original MRI report, postoperative histopathology, treatment data, and the other reader’s classifications and were aware only that the examinations had been obtained because of penile cancer. Inter-reader agreement was assessed in the full cohort (*n* = 38), and no imputation was performed. By contrast, nodal diagnostic accuracy against pathological nodal status remained restricted to the 30 patients with complete pathological verification.

### 2.4. Reference Standard (Histopathology)

The reference standard for local staging was the final postoperative histopathology (pT). The histopathological T categories in this cohort included Tis/Ta, T1a, T1b, T2, and T3. For nodal staging, the reference standard was pathological nodal status (pN) obtained by complete pathological verification. In cN0 patients for whom invasive staging was indicated, verification was obtained using dynamic sentinel node biopsy (DSNB); in cN+ patients, verification was obtained by radical inguinal lymphadenectomy. Complete pathological nodal verification was available in 30 patients. The remaining eight patients had cN0 groins and low-risk primary tumours according to EAU criteria; invasive nodal staging was therefore not indicated [12]. These eight patients were excluded from the N-stage diagnostic-accuracy analysis because no pathological nodal reference standard was available. This guideline-concordant risk-based verification strategy was considered when interpreting the nodal results.

## 3. Statistical Analysis

Diagnostic performance was assessed using contingency tables, reporting accuracy, sensitivity, specificity, positive predictive value (PPV), and negative predictive value (NPV), each with 95% confidence intervals (CIs) calculated using the Wilson score method. Agreement between the original MRI classification and histopathology was quantified using Cohen’s κ for binary endpoints and both unweighted and quadratic-weighted κ for the three-category endpoint. Systematic directional disagreement was evaluated using McNemar’s test (two-sided α = 0.05).

Because the MRI index test generated categorical stage assignments rather than continuous probability scores, conventional receiver operating characteristic curves and areas under the curve were not reported. For binary local and nodal staging, balanced accuracy was calculated as the arithmetic mean of sensitivity and specificity. Ninety-five percent CIs for balanced accuracy were estimated using the Wald approximation based on the binomial variances of sensitivity and specificity.

Inter-reader agreement was reported as exact percentage agreement and Cohen’s κ with 95% CIs for binary T-stage and N-stage classifications. For the three-category ordinal T-stage classification, both unweighted and quadratic-weighted κ were reported.

Exploratory associations between correct versus incorrect MRI classification and tumour grade or HPV status were assessed separately for local T staging (*n* = 38) and nodal staging (*n* = 30) using Pearson’s chi-squared tests with simulated *p*-values. Lymphovascular invasion, perineural invasion, and detailed histological-subtype comparisons were not analysed because these variables were not systematically captured as independent structured variables, and no distinct subgroup of a rare invasive histological variant was present. No formal a priori sample-size calculation was performed because the study included the entire consecutive cohort of eligible MRI-examined patients available during the prespecified period. Accordingly, the primary emphasis was placed on diagnostic-performance estimates and their 95% confidence intervals rather than on achieving statistical significance. To inform the design of a future larger validation study, an illustrative precision-based sample-size calculation was performed using the conventional single-proportion approach for sensitivity and specificity, adjusted for disease prevalence. The calculation assumed a pooled sensitivity of 0.86 and specificity of 0.89, as reported in the previous meta-analysis [17], the observed prevalence of ≥T2 disease in the present cohort (17/38; 44.7%), a two-sided 95% confidence level, and a target absolute confidence-interval half-width of 0.10. The larger of the sensitivity- and specificity-based requirements corresponded to approximately 104 evaluable patients; after allowing for 10% incomplete data, the corresponding enrolment target would be approximately 116 patients. This calculation was performed solely to inform future study planning and was not used to determine or retrospectively justify the size of the present cohort. Subgroup analyses were considered exploratory because non-significant findings may reflect a type II error. Missing data were not imputed for the primary diagnostic-accuracy analyses. All statistical analyses were performed using R software (version 4.3.2).

### Sensitivity Analysis for Equivocal T1/T2 Cases

An exploratory sensitivity analysis evaluated the impact of four identified equivocal MRI cases with borderline features between T1 and T2 (10.5% of the cohort). In the primary (conservative) approach, these cases were retained as MRI ≥ T2, reflecting the original clinical classification of the disease. In the alternative (optimistic) scenario, all four cases were reclassified as MRI ≤ T1, and all diagnostic metrics were recalculated to assess robustness.

## 4. Results

Table 1 presents a summary of the baseline clinicopathological characteristics of the patients. The median age of the cohort was 65 years (IQR 54.5–71.8). All tumours were located in the glans, affecting 38 (100%) patients in this study. Definitive surgical interventions involved organ-sparing procedures in all 38 (100%) patients. The distribution of histopathological T-stages was as follows: pTis in 4 (10.5%) patients, pT1a in 12 (31.6%), pT1b in 5 (13.2%), pT2 in 11 (28.9%), and pT3 in 6 (15.8%) patients. Complete pathological nodal verification was available for 30/38 patients (78.9%). In the verified N-staging subset, cN0 patients underwent DSNB and cN+ patients underwent radical inguinal lymphadenectomy as the reference-standard nodal procedure. Pathology revealed pN0 disease in 16/30 (53.3%) and pN+ disease in 14/30 (46.7%) patients. The remaining 8/38 patients (21.1%) had no complete pathological nodal verification and were therefore excluded from the N-staging diagnostic accuracy analysis.

### 4.1. Cohort Size and T-Stage Distributions

A total of 38 patients were included in the T-staging analysis. Histopathological examination showed that 21/38 (55.3%) patients had ≤T1 disease and 17/38 (44.7%) had ≥T2 disease. On MRI, the proportion classified as advanced stage was higher: 25/38 (65.8%) were classified as ≥T2, indicating a tendency to over-stage relative to histopathology findings.

### 4.2. Primary Endpoint: Binary T-Staging Accuracy (≤T1 vs. ≥T2)

**Figure 6:**
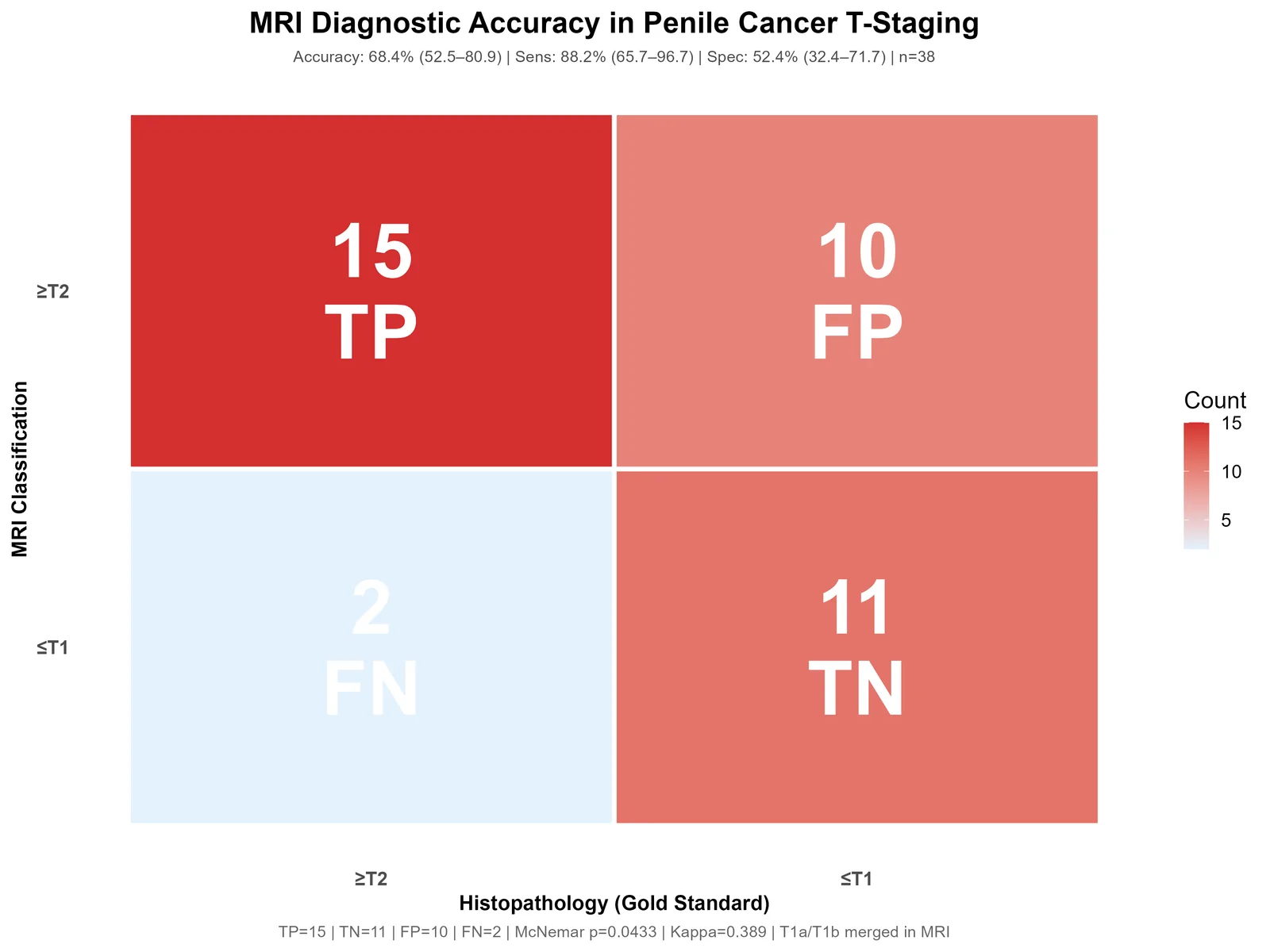
Binary local T-stage confusion matrix for MRI versus postoperative histopathology (≤T1 vs. ≥T2). Rows represent MRI classification, and columns represent the histopathological reference standard. MRI detected most pathologically advanced tumours (TP = 15; FN = 2) but frequently overstaged early disease (FP = 10).

**Figure 7:**
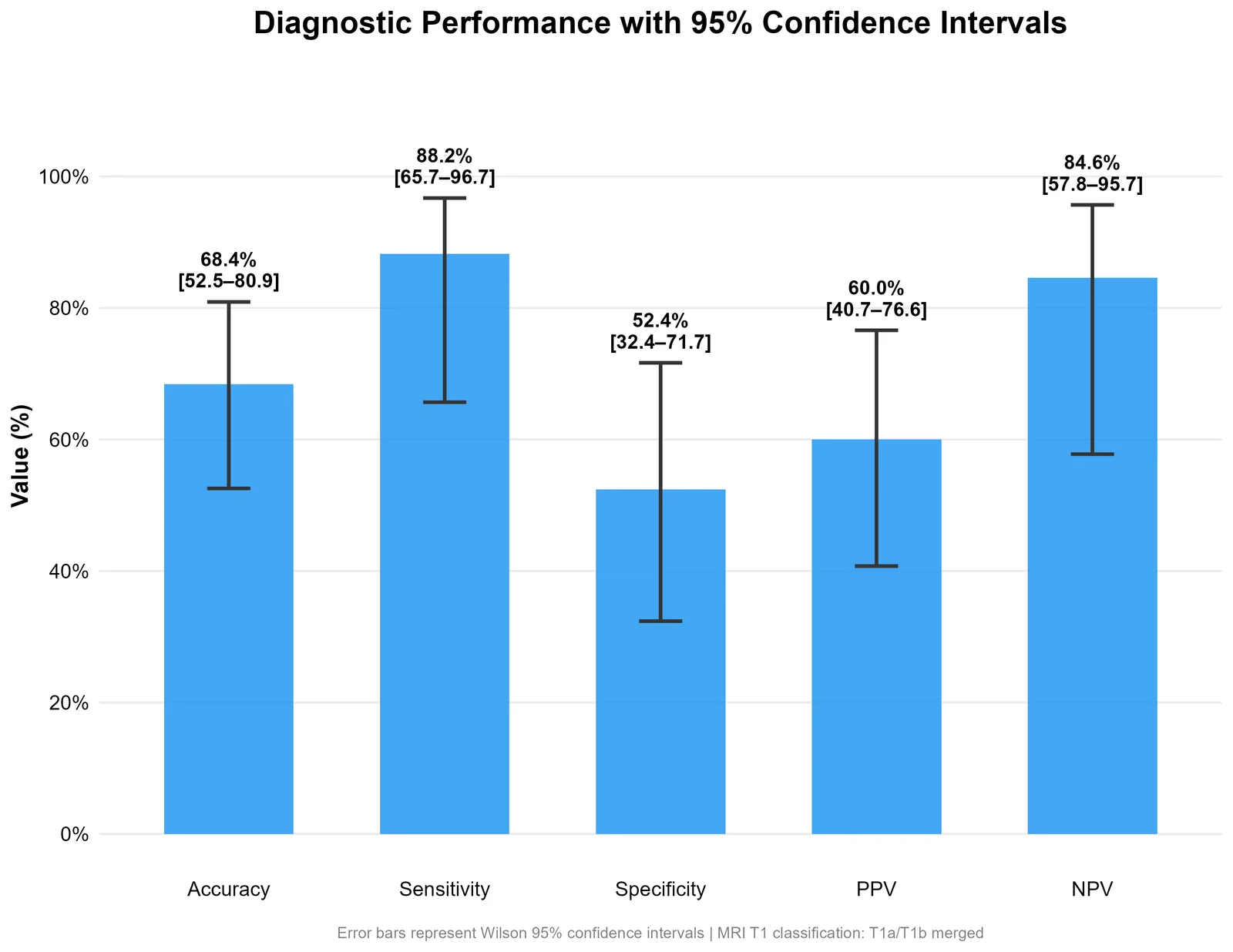
Diagnostic performance of MRI for binary local T-staging with 95% confidence intervals. Sensitivity was high, whereas specificity and PPV were limited, consistent with the tendency towards overstaging.

For binary T-stage classification, MRI achieved an accuracy of 68.4% (26/38; 95% confidence interval [CI], 52.5–80.9%). The sensitivity for detecting ≥T2 disease was 88.2% (15/17; 95% CI 65.7–96.7%), and the specificity was 52.4% (11/21; 95% CI 32.4–71.7%). PPV was 60.0% (15/25; 95% CI 40.7–76.6%) and NPV was 84.6% (11/13; 95% CI 57.8–95.7%). The corresponding confusion matrix and summary diagnostic metrics are presented in Figure 6 and Figure 7, respectively.

The corresponding confusion matrix comprised 15 true-positive classifications, defined as MRI ≥ T2 and histopathology ≥T2; 11 true-negative classifications, defined as MRI ≤ T1 and histopathology ≤T1; 10 false-positive classifications, defined as MRI ≥ T2 but histopathology ≤T1; and two false-negative classifications, defined as MRI ≤ T1 but histopathology ≥T2 (Figure 6). The agreement between MRI and histopathology for binary T-staging was fair (Cohen’s κ = 0.389). There was evidence of systematic overstaging, with more false-positive than false-negative classifications (FP = 10 vs. FN = 2; McNemar’s test with continuity correction, *p* = 0.043). An exploratory reclassification of the four equivocal T1/T2 cases did not change overall accuracy (68.4%) but reduced sensitivity from 88.2% to 76.5% and increased specificity from 52.4% to 61.9%, demonstrating the expected sensitivity–specificity trade-off. The complete results are provided in Supplementary Table S1.

### 4.3. Secondary Endpoint: Three-Category T-Staging Agreement (≤T1/T2/≥T3)

**Figure 8:**
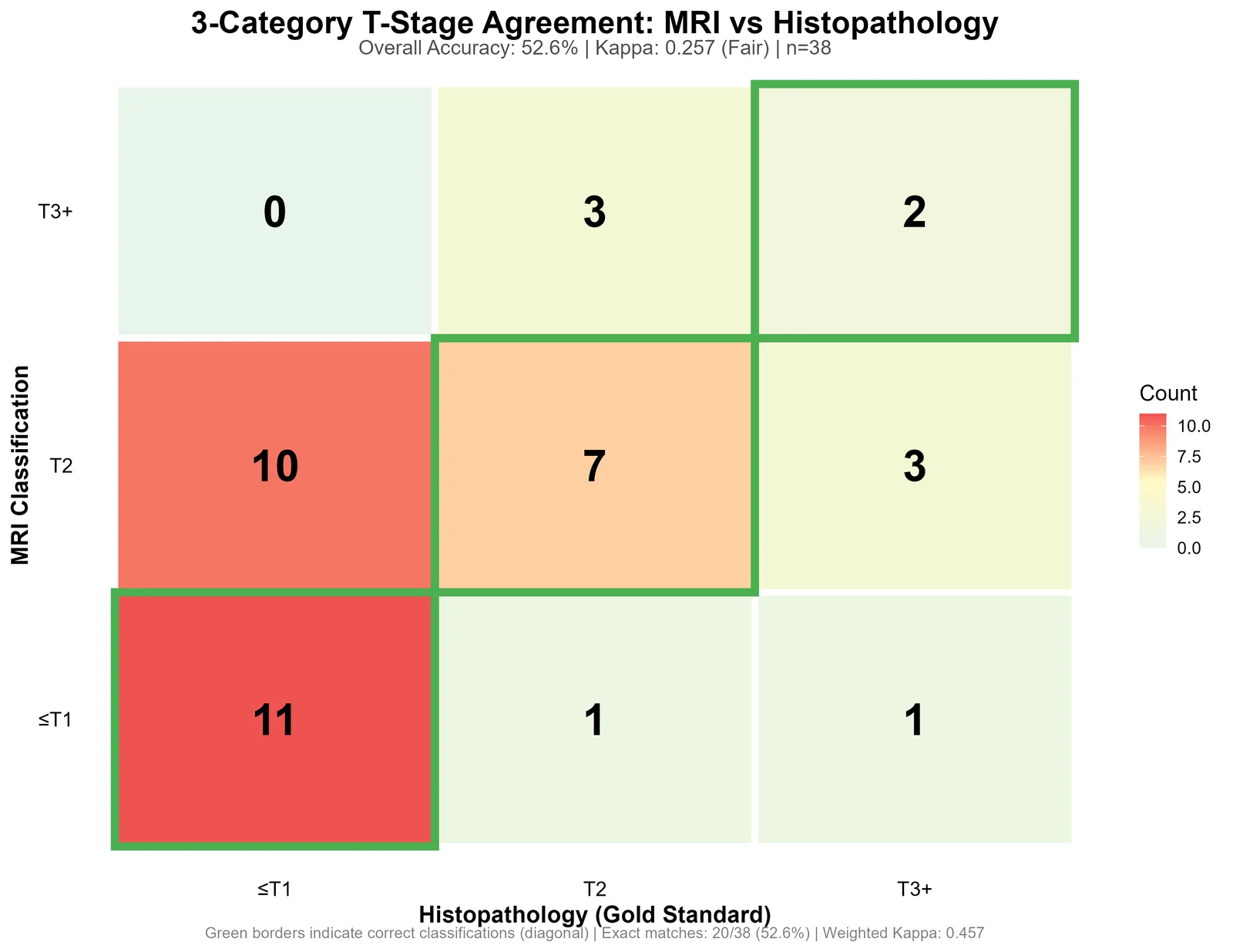
Three-category local T-stage agreement between MRI and histopathology (≤T1, T2, and T3+). Misclassification was clustered at the T1/T2 and T2/T3 interfaces, with frequent overclassification of ≤T1 as T2 and understaging of pT3+ as T2.

For the three-category T-stage endpoint, MRI demonstrated an overall exact agreement of 52.6% (20/38) with fair agreement (Cohen’s κ = 0.257). When the ordinal nature of the three-category classification was considered, the quadratic-weighted agreement was higher but remained limited (weighted κ = 0.457). The 3 × 3 contingency table showed frequent misclassifications of ≤T1 as T2 (10 cases); the complete agreement matrix is presented in Figure 8.

Advanced-stage discrimination was limited. Among the six pathologically confirmed ≥T3 tumours, MRI classified two (33.3%) as ≥T3, three (50.0%) as T2, and one (16.7%) as ≤T1.

In a one-vs.-rest analysis, the sensitivity and specificity were as follows:

• ≤T1: sensitivity 52.4%, specificity 88.2%;
• T2: sensitivity 63.6%, specificity 51.9%;
• ≥T3: sensitivity 33.3%, specificity 90.6%.

Importantly, misclassification in the three-category analysis was not random but clustered at clinically relevant decision-making points. Early-stage tumours were frequently overstaged as T2, whereas most pT3+ tumours were understaged as T2, indicating that MRI had a limited ability to resolve the T1/T2 and T2/T3 interfaces in this cohort.

### 4.4. Nodal Staging (N0 vs. N+)

**Figure 9:**
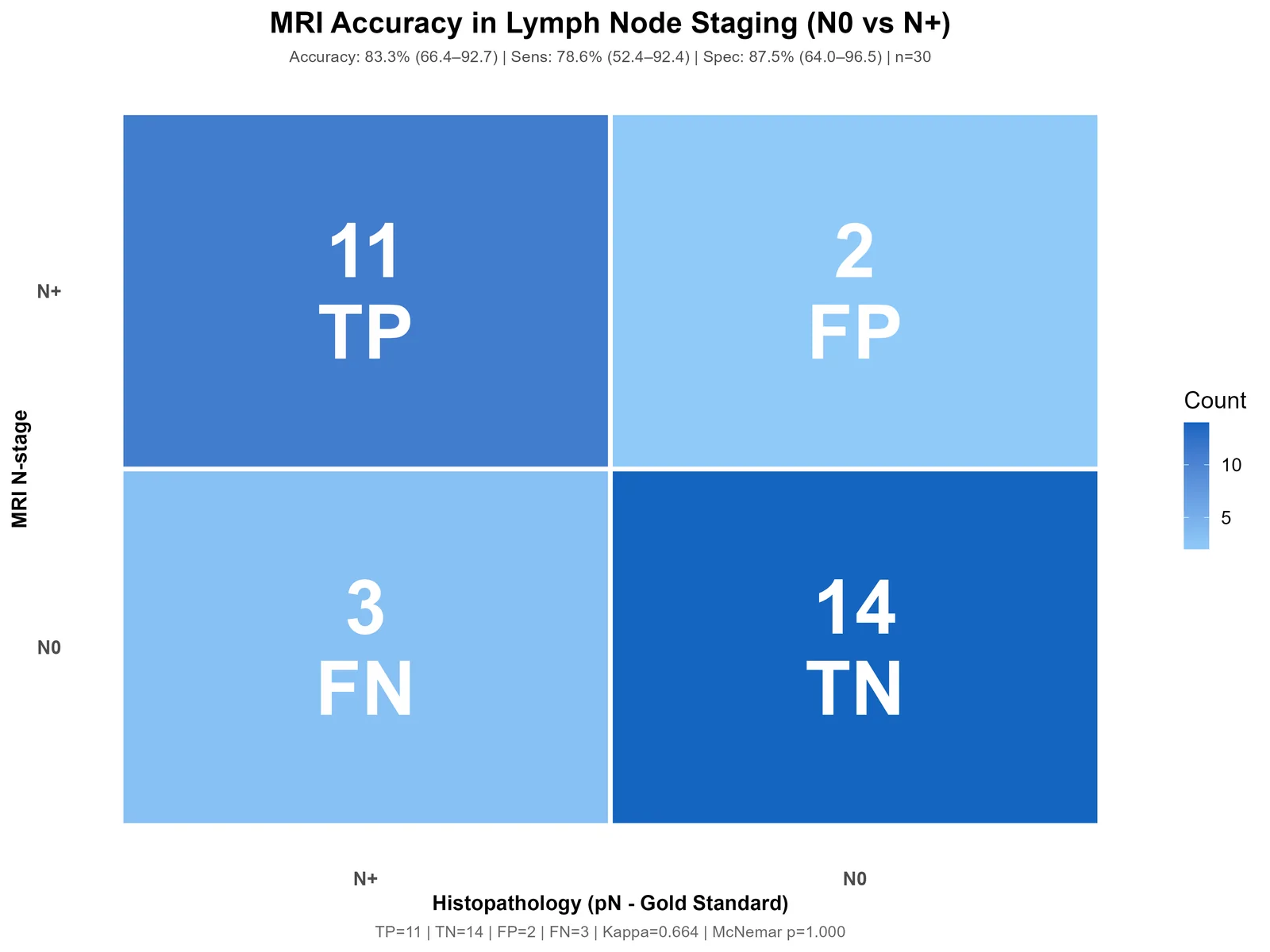
Binary nodal staging (N0 vs. N+) by MRI versus pathological nodal status in patients with complete pathological nodal verification (*n* = 30). Rows represent MRI classification, and columns represent the pathological reference standard. Verification was obtained using DSNB in cN0 patients with an indication for invasive staging and radical inguinal lymphadenectomy in cN+ patients. Eight cN0 patients with EAU low-risk primary tumours did not undergo invasive nodal staging and were not included in this analysis.

Complete pathological nodal verification was available for 30/38 patients. The remaining eight patients had cN0 groins and EAU low-risk primary tumours; invasive nodal staging was not indicated, and they were excluded from this endpoint because no pathological nodal reference standard was available. In the verified subset, cN0 patients underwent DSNB and cN+ patients underwent radical inguinal lymphadenectomy. The pN+ prevalence was 46.7% (14/30), indicating that this was a guideline-selected verified subset rather than an unselected cN0 population. For MRI N staging (N0 vs. N+), accuracy was 83.3% (25/30; 95% CI, 66.4–92.7%), sensitivity was 78.6% (11/14; 95% CI, 52.4–92.4%), specificity was 87.5% (14/16; 95% CI, 64.0–96.5%), PPV was 84.6% (11/13; 95% CI, 57.8–95.7%), and NPV was 82.4% (14/17; 95% CI, 59.0–93.8%). The confusion matrix comprised TP = 11, TN = 14, FP = 2, and FN = 3, with Cohen’s κ = 0.664, McNemar *p* = 1.000, and balanced accuracy of 0.830 (95% CI, 0.691–0.970) (Figure 9). Because PPV and NPV depend on disease prevalence and verification was risk-based, these predictive values apply specifically to the pathologically verified subset and should not be extrapolated directly to an unselected cN0 population.

The diagnostic performance of MRI across the primary binary local T-staging endpoint, three-category local T-staging endpoint, and binary nodal staging endpoint is summarised in Table 2 and Table 3. For the primary binary endpoint (≤T1 vs. ≥T2), MRI demonstrated high sensitivity but limited specificity, resulting in a significant predominance of false-positive over false-negative classifications. Exact agreement was lower for three-category local T-staging, whereas binary nodal staging showed higher overall accuracy in the histopathologically verified node subset.

### 4.5. Post Hoc Inter-Reader Reproducibility

In the all-case analysis (*n* = 38), exact inter-reader agreement for binary local T staging was 92.1% (35/38), with κ = 0.837 (95% CI, 0.719–0.954). For three-category local T staging, exact agreement was 89.5% (34/38), with unweighted κ = 0.825 (95% CI, 0.705–0.946) and quadratic-weighted κ = 0.734 (95% CI, 0.593–0.874). For binary N staging, exact agreement was 97.4% (37/38), with κ = 0.943 (95% CI, 0.869–1.000) (Table 4).

### 4.6. Exploratory Clinicopathological Subgroup Analyses

No statistically significant association was observed between correct local T-stage classification and tumour grade (*p* = 0.9645) or HPV status (*p* = 0.2444). Similarly, no statistically significant association was observed between correct nodal classification and tumour grade (*p* = 0.8251) or HPV status (*p* = 1.000). Given the small subgroup sizes, these findings do not establish equivalent or subgroup-independent MRI performance and should be regarded as exploratory.

## 5. Discussion

In this single-centre diagnostic accuracy study, preoperative mpMRI demonstrated moderate performance in binary local T staging of penile cancer. MRI detected most pathologically advanced tumours with a sensitivity of 88.2% and a favourable negative predictive value of 84.6%. However, the specificity was limited to 52.4%, and a paired comparison with histopathology showed significant directional disagreement towards overstaging. Taken together, these findings indicate that MRI is better at minimising the risk of missed ≥T2 disease than at confidently confirming an advanced local stage. Accordingly, a positive MRI result suggesting ≥T2 disease should not be interpreted as definitive in isolation.

In 2021, Flammia et al. presented one of the first systematic reviews and meta-analyses on the diagnostic accuracy of MRI in the preoperative local staging of penile cancer. Based on seven studies comprising 430 patients, the authors analysed the accuracy of MRI in detecting pathologic T-stage ≥ T2, showing a sensitivity of 86% and specificity of 70% [19]. Another important contribution, particularly from the perspective of evaluating the utility of an artificially induced erection MRI protocol, was the meta-analysis by Krishna et al. in 2022 [17]. Drawing on eight studies with a combined total of 481 patients, the authors confirmed a comparable sensitivity (86%) with a higher specificity (89%) [17]. Furthermore, Krishna et al. demonstrated that the use of artificially induced erection did not significantly improve the diagnostic accuracy of MRI in local staging and that MRI did not outperform clinical staging in local T stage assessment [17]. Although the sensitivity of 88.2% in our study is consistent with these findings, a notable discrepancy was observed in the markedly lower specificity observed in our cohort (52.4%). This lower specificity should be interpreted cautiously and may reflect the small sample size, case-mix variation, exclusive predominance of glans tumours treated with organ-sparing surgery, differences in MRI interpretation thresholds, and the conservative classification of equivocal T1/T2 findings as MRI ≥ T2 in the primary analysis. The median BMI of 29.0 kg/m^2^ and the 44.7% prevalence of phimosis describe this selected tertiary-centre MRI cohort and should not be interpreted as population prevalence estimates. Phimosis may impede physical examination and may have contributed to referral for additional imaging, although this could not be formally tested. These characteristics may therefore reflect referral and spectrum selection. These findings support the role of MRI as a complementary tool in the preoperative local staging of penile cancer, to be interpreted in conjunction with clinical examination and histopathological assessment, rather than serving as a stand-alone basis for therapeutic decision-making.

Given the functional and psychosocial significance of the penis, accurate treatment planning for penile cancer is essential. Current management strategies aim to achieve complete tumour resection with negative oncological margins while maximising organ preservation whenever feasible [12,20]. As treatment selection is strongly dependent on the local tumour stage, inaccuracies in preoperative staging may lead to inappropriate escalation or de-escalation of therapy, including unnecessary radical surgery or invasive nodal staging procedures. Although MRI does not outperform clinical examination in distinguishing T1 from T2 disease, current guidelines support its use in selected clinical scenarios, including diagnostic uncertainty regarding corporal body invasion, assessment of the resectability of large tumours, and preoperative planning of organ-sparing surgery [12,17]. In 2021, Ghosh et al. reported a high overall concordance between MRI and histopathology in a cohort of 54 patients examined without an artificially induced erection protocol [21]. However, both over- and understaging errors were observed, leading the authors to conclude that no predominant pattern of staging discrepancies was present. Similar observations were reported by Iqbal et al. in a retrospective study including patients examined both with and without prostaglandin E1-induced artificial erection [22]. The authors observed comparable numbers of over- and understaging errors for T1 and T2 diseases and found no diagnostic advantage associated with the artificially induced erection protocol. A different pattern was observed by Kayes et al., who reported a predominance of overstaging errors in 55 patients, with most discrepancies occurring at the T1/T2 interface [23]. These findings are consistent with the dominant tendency toward overstaging observed in our cohort (FP = 10 vs. FN = 2, *p* = 0.043). This asymmetry is further reflected in the PPV of 60.0%, indicating that 10 of 25 MRI-positive classifications (40.0%) were not confirmed as ≥T2 on final histopathological examination.

Several factors may contribute to the observed tendency of overstaging. Peritumoural oedema, inflammatory reactions, tumour surface irregularities, and imaging artefacts may mimic deeper stromal invasion and generate false-positive findings [14,22,23,24]. Because the timing of biopsy or cytological sampling relative to MRI was not consistently recorded, post-procedural haemorrhage, oedema, inflammation, or architectural distortion may also have contributed to false-positive ≥T2 classifications; the magnitude of this effect could not be quantified. A further structural limitation, particularly relevant to decisions regarding invasive nodal staging, is the inability of MRI to distinguish pT1a from pT1b disease. Under the current TNM classification, this distinction depends on lymphovascular invasion, perineural invasion, and grade, which are histopathological features beyond the resolving capacity of imaging [25,26]. Thus, MRI findings suggestive of ≥T2 disease should not independently drive treatment escalation and must be interpreted with clinical and histopathological findings. When the analysis was extended beyond the binary endpoint of distinguishing early from advanced disease to the three-category classification of ≤T1, T2, and ≥T3, the diagnostic performance declined markedly. Although the binary classification yielded an accuracy of 68.4% and fair agreement with histopathology (κ = 0.389), the three-category analysis showed exact agreement in only 52.6% of cases (κ = 0.257). Misclassification was not randomly distributed but clustered predominantly at clinically relevant stage boundaries; early-stage tumours were frequently overstaged as T2, while pT3+ disease was commonly understaged as T2. This was particularly evident for advanced tumours: among six pathologically confirmed ≥T3 tumours, MRI classified two (33.3%) as ≥T3, three (50.0%) as T2, and one (16.7%) as ≤T1. These findings are consistent with the broader observation that MRI performs less reliably in precise local staging than in addressing the simpler question of whether an advanced disease is present. Ghosh et al. and Switlyk et al. reported weighted κ values of 0.85 and 0.742 for overall T-staging, although both studies reported residual discrepancies at key stage boundaries [21,27]. Iqbal et al. similarly noted that staging errors were most prevalent for T1 and T2 diseases, with performance improving primarily for cases with clear corporal invasion [22]. These observations collectively suggest that the diagnostic value of MRI in penile cancer lies primarily in identifying or excluding advanced local disease rather than in providing precise anatomical stage assignment, a distinction with direct implications for how MRI findings should be weighted in multidisciplinary treatment planning.

Because nodal status is the dominant prognostic determinant in penile cancer, guideline-concordant invasive nodal staging remains essential [28]. Imaging cannot reliably detect micrometastatic disease in clinically node-negative patients and therefore cannot replace DSNB. Its role is complementary: although bulky inguinal disease is usually clinically apparent, cross-sectional imaging may help define the full regional extent of nodal disease, identify pelvic nodal involvement, assess extranodal extension, and support operative or multimodal treatment planning [12]. In a study by Lucchesi et al., MRI correctly staged nodal disease in 13 of 15 patients (86.7%), compared with 46.7% for clinical examination, although false-positive findings occurred because of reactive inguinal lymphadenopathy [29]. Krishna et al. likewise concluded that MRI may improve assessment of macroscopic inguinal and pelvic nodal disease but also stressed that small nodal metastases and differentiation between reactive and metastatic enlarged lymph nodes remain problematic [30]. Our findings are consistent with this concept. In the 30 patients with reference-standard pathological nodal verification, MRI achieved an accuracy of 83.3%, sensitivity of 78.6%, specificity of 87.5%, PPV of 84.6%, NPV of 82.4%, substantial agreement with pathology (κ = 0.664), and balanced accuracy of 0.830 (95% CI, 0.691–0.970). This N0/N+ performance is clinically encouraging and suggests that MRI may provide useful macroscopic nodal information for preoperative planning, particularly when integrated with physical examination and guideline-based nodal assessment. However, because MRI cannot reliably exclude micrometastatic disease and because this nodal analysis was limited to the pathologically verified subset, these results should not imply that MRI can replace DSNB in cN0 high-risk patients or radical inguinal lymphadenectomy in cN+ disease.

Clinically, our findings do not support routine MRI for local staging in all patients with penile cancer, nor do they demonstrate a clear incremental value for an artificial-erection MRI protocol. As this study did not include a direct comparator group undergoing MRI without artificial erection, it could not determine whether artificial erection is unnecessary. Nevertheless, the limited specificity, frequent overstaging, and poor three-category agreement suggest that MRI should be reserved for selected staging dilemmas, such as equivocal clinical assessment of corporal invasion or situations in which imaging may influence organ-preserving surgical planning.

MRI performance may differ in patients with bulky or more advanced tumours undergoing partial or total penectomy, because frank corpus cavernosum, urethral, or adjacent-organ invasion may be more conspicuous than the subtle T1/T2 interfaces that predominated in this cohort. Such cases were not adequately represented, and the present estimates should not be extrapolated directly to that population. MRI may support assessment of organ-preserving feasibility and the anticipated extent of penectomy, but it should not independently determine the procedure. Treatment selection must integrate physical examination, biopsy histology, tumour size and location, the ability to achieve negative margins, anticipated residual functional penile length, patient preference, and multidisciplinary assessment [12]. Given the observed low specificity and tendency towards overstaging, escalation based on MRI alone could result in overtreatment.

The strengths of this study include the use of postoperative histopathology as the reference standard, assessment of both local and nodal staging endpoints, inclusion of consecutive eligible patients undergoing preoperative MRI, transparent reporting of diagnostic measures with confidence intervals, and an independent two-reader reassessment. However, this study has several limitations. First, the cohort was small and sourced from a single tertiary centre, resulting in wide confidence intervals and limited external validity. No formal a priori sample-size calculation was performed because all consecutive eligible MRI-examined patients available during the prespecified period were included. The precision-based calculation reported in the Methods was intended solely to inform the design of a future larger validation study. Accordingly, the present findings should be interpreted together with their 95% confidence intervals, and the subgroup analyses should remain exploratory. Second, the inclusion of only surgically treated patients may have introduced spectrum and verification biases. Third, complete pathological nodal verification was available for only 30 of 38 patients; thus, nodal staging results may not be generalisable to all clinically node-negative patients. Moreover, the verified nodal subset combined cN0 patients undergoing DSNB and cN+ patients undergoing radical inguinal lymphadenectomy, resulting in a relatively high prevalence of pN+ disease; therefore, PPV and NPV should not be extrapolated directly to an unselected cN0 population. Fourth, the inter-reader analysis was post hoc and involved two radiologists from the same specialist centre; high agreement may not generalise to less experienced readers or external institutions. Fifth, the exact biopsy-to-MRI interval was not consistently available, and the influence of post-procedural changes could not be quantified. Sixth, subgroup analyses were exploratory and underpowered; p16 was used only as a surrogate marker because direct HPV DNA or RNA testing was not performed, and lymphovascular and perineural invasion were not systematically available as structured variables. Finally, the low specificity and significant tendency towards overstaging mean that MRI-positive findings for ≥T2 disease should be interpreted cautiously and should not be used alone to escalate treatment.

## 6. Conclusions

Preoperative multiparametric MRI demonstrated high sensitivity but limited specificity for distinguishing ≤T1 from ≥T2 disease and showed a significant tendency towards overstaging. Precise three-category local staging was less reliable, particularly at the T1/T2 and T2/T3 interfaces. The post hoc two-reader reassessment showed high inter-reader agreement, suggesting that the observed limitations of precise local staging were not solely attributable to reader variability. These findings apply primarily to a small cohort of patients with glans-based tumours treated by organ-sparing surgery. Because no non-erection MRI comparator was included, the study does not establish an incremental diagnostic benefit of pharmacologically induced erection. Binary nodal staging showed encouraging performance in the 30 patients with pathological verification. Eight cN0 patients with EAU low-risk primary tumours appropriately did not undergo invasive nodal staging. Nevertheless, the risk-based verification strategy and high pN+ prevalence in the verified subset limit generalisability of the predictive values. MRI may support selected local-staging and operative-planning decisions and may help define the extent of macroscopic nodal disease, but it cannot replace clinical examination, histopathological assessment, indicated DSNB, or therapeutic lymphadenectomy. Larger multicentre studies with standardised acquisition protocols, prespecified independent readers, and external validation are required.

## Figures and Tables

**Table 1:** Baseline clinical and histopathological characteristics of the study cohort.

Characteristic	Value
**Clinical and demographic characteristics**	
Total cohort	38 patients
Age (years), (IQR); range	65.0 (54.5–71.8); 34–82
BMI, kg/m^2^, (IQR); range	29.0 (26.3–30.8); 22–38
Phimosis	17 (44.7%)
HPV-positive status	8 (21.1%)
**Histopathological characteristics **	
PeIN/pTis	4 (10.5%)
Squamous cell carcinoma usual type	34 (89.5%)
**Tumour grade among invasive squamous cell carcinomas (*n* = 34) **	
G1	11 (32.4%)
G2	15 (44.1%)
G3	8 (23.5%)
**Pathological local T stage **	
pTis	4 (10.5%)
pT1a	12 (31.6%)
pT1b	5 (13.2%)
pT2	11 (28.9%)
pT3	6 (15.8%)
** Binary pathological T-stage endpoint **	
≤T1	21 (55.3%)
≥T2	17 (44.7%)
** Pathological nodal assessment **	
Complete pathological nodal verification	30/38 (78.9%)
pN0 in verified N-staging subset	16/30 (53.3%)
pN+ in verified N-staging subset	14/30 (46.7%)
** Follow-up and outcomes **	
Postoperative follow-up/survival (months); (IQR); range	31.0 (14.3–45.8); 2–93
Deaths during follow-up	11 (28.9%)
Penile cancer-related deaths	10 (26.3%)
Non-penile cancer deaths	1 (2.6%)

Note: Data are presented as *n* (%) unless otherwise stated. Percentages were calculated using the total cohort as the denominator (*n* = 38), except for pN status, which was calculated among patients with complete pathological nodal verification (*n* = 30). In cN0 patients, nodal verification was performed using dynamic sentinel node biopsy (DSNB) and, in cN+ patients, using radical inguinal lymphadenectomy. Tumour-grade percentages were calculated among the 34 invasive usual-type squamous cell carcinomas. p16 positivity was used as a surrogate marker of HPV association; direct HPV DNA or RNA testing was not performed. BMI, body mass index; IQR, interquartile range.

**Table 2:** Diagnostic performance of preoperative MRI for local and nodal staging of penile cancer.

Metric	Binary Local T-Stage ≤T1 vs. ≥T2	Three-Category Local T-Stage ≤T1/T2/≥T3	Binary Nodal Stage N0 vs. N+
analysis set, *n*	38	38	30
reference-positive category	histopathology ≥ T2	not applicable	pathological N+
MRI-positive category	MRI ≥ T2	not applicable	MRI N+
confusion matrix	TP = 15; TN = 11; FP = 10; FN = 2	see 3 × 3 cross-tabulation below	TP = 11; TN = 14; FP = 2; FN = 3
accuracy/exact agreement, % (95% CI)	68.4 (52.5–80.9)	52.6 (20/38)	83.3 (66.4–92.7)
sensitivity, % (95% CI)	88.2 (65.7–96.7)	—	78.6 (52.4–92.4)
specificity, % (95% CI)	52.4 (32.4–71.7)	—	87.5 (64.0–96.5)
PPV, % (95% CI)	60.0 (40.7–76.6)	—	84.6 (57.8–95.7)
NPV, % (95% CI)	84.6 (57.8–95.7)	—	82.4 (59.0–93.8)
Cohen ’ s κ	0.389	0.257	0.664
quadratic-weighted κ	—	0.457	—
balanced accuracy	0.703 (95% CI 0.568–0.838)	—	0.830 (95% CI, 0.691–0.970)
bias/asymmetry test	FP = 10 vs. FN = 2; McNemar *p* = 0.043	not assessed	FP = 2 vs. FN = 3; McNemar *p* = 1.000

**Table 3:** Three-category local T-stage cross-tabulation.

MRI Category	pT ≤ T1	pT2	pT ≥ T3	Total
MRI ≤ T1	11	1	1	13
MRI T2	10	7	3	20
MRI ≥ T3	0	3	2	5
Column total	21	11	6	38

Note: CI: confidence interval; FN: false negative; FP: false positive; MRI: magnetic resonance imaging; NPV: negative predictive value; PPV: positive predictive value; TN: true negative; TP: true positive. For binary local T staging, MRI ≥ T2 was considered positive, and histopathology ≥T2 was the reference. For binary nodal staging, MRI N+ was considered positive, and pathological N+ was considered the reference. The pN status was established using DSNB in cN0 patients and radical inguinal lymphadenectomy in cN+ patients. Confidence intervals for proportions were calculated using the Wilson method.

**Table 4:** Post hoc inter-reader reproducibility of MRI staging.

Endpoint	Analysis Set, *n*	Exact Agreement, *n* (%)	Cohen’s κ (95% CI)	Quadratic-Weighted κ (95% CI)
Binary local T stage (≤T1 vs. ≥T2)	38	35/38 (92.1%)	0.837 (0.719–0.954)	—
Three-category local T stage (≤T1/T2/≥T3)	38	34/38 (89.5%)	0.825 (0.705–0.946)	0.734 (0.593–0.874)
Binary N stage (N0 vs. N+)	38	37/38 (97.4%)	0.943 (0.869–1.000)	—

Note: Agreement estimates were calculated from paired reader-level classifications for all 38 examinations; no imputation was performed.

## Data Availability

De-identified clinical, imaging, and histopathological data underlying the main analyses, together with analytic code, can be made available upon reasonable request to the corresponding author, subject to institutional approval and signing of a data use agreement.

## Supplementary Materials

The following supporting information can be downloaded at: https://www.mdpi.com/article/10.3390/cancers18162674/s1, Table S1. Exploratory sensitivity analysis for equivocal T1/T2 MRI classifications.

## Abbreviations

The following abbreviations are used in this manuscript:

MRI	Magnetic Resonance Imaging
DSNB	Dynamic Sentinel Node Biopsy
CI	Confidence Interval
PPV	Positive Predictive Value
NPV	Negative Predictive Value
FP	False Positive
FN	False Negative
USA	The United States of America
HPV	Human Papillomavirus
EAU	European Association of Urology
ASCO	American Society of Clinical Oncology
WHO	World Health Organization
mpMRI	Multiparametric Magnetic Resonance Imaging
DWI	Diffusion-Weighted Imaging
DCE	Dynamic Contrast-Enhanced
IQR	Interquartile Range
ESUR	European Society of Urogenital Radiology
ADC	Apparent Diffusion Coefficient
pT	Pathological T-stage
pN	Pathological N-stage
BMI	Body Mass Index
PeIN	Penile Intraepithelial Neoplasia
TP	True Positive
TN	True Negative
TNM	Tumour, Node, Metastasis classification
