# Supplementary material for: Diagnostic Performance of Preoperative Multiparametric MRI for Local and Nodal Staging in Penile Cancer: A Histopathology-Correlated Single-Centre Study
Source: Cancers (Basel). 2026 Aug 18;18(16):2674. doi: 10.3390/cancers18162674 (PMC13510312; doi:10.3390/cancers18162674)
Supplement: Supplementary file 1 [file cancers-18-02674-s001.zip › cancers-4477486-supplementary.pdf]

Table S1. Exploratory sensitivity analysis for equivocal T1/T2 MRI classifications.

Scenario	TP	TN	FP	FN	Accuracy	Sensitivity	Specificity	PPV	NPV	κ / balanced accuracy / McNemar p
Primary original-report classification	15	11	10	2	68.4%	88.2%	52.4%	60.0%	84.6%	κ = 0.389; balanced accuracy = 0.703; p = 0.043
Alternative: four equivocal cases reclassified as ≤T1	13	13	8	4	68.4%	76.5%	61.9%	61.9%	76.5%	κ = 0.375; balanced accuracy = 0.692; p = 0.386
